# Adherence to 24-hour movement guidelines among rural Brazalian preschool children: associations with parenting practices

**DOI:** 10.1186/s12966-022-01369-y

**Published:** 2022-10-21

**Authors:** Widjane Sheila Ferreira Goncalves, Rebecca Byrne, Pedro Israel Cabral de Lira, Marcelo Tavares Viana, Stewart G. Trost

**Affiliations:** 1 School of Exercise and Nutrition Sciences, Queensland University of Technology, Brisbane, Australia; 2 Department of Nutrition, Federal University of Pernambuco, Recife, PE Brazil; 3 Department of Health Sciences, Federal University of Pernambuco, Recife, PE Brazil; 4 School of Human Movement and Nutrition Sciences, The University of Queensland, Brisbane, Australia

**Keywords:** Parenting, Physical activity, Screen time, Sedentary behavior, Sleep, Children

## Abstract

**Background:**

Young children residing in rural areas of low-and-middle income countries (LMICs) such as Brazil are at greater risk of obesity and related chronic health conditions. Yet, the extent to which rural preschool children from Brazil aged 3- to 6-years meet the World Health Organisation (WHO) 24-hour movement guidelines is unknown. Parents play a central role in the development of children’s movement behaviors with logistic support, co-participation, modelling, and setting rules and limits recognized as influential parenting practices. However, the bulk of parenting research has been conducted in families from high income countries (HICs) and little is known about the relationship between parenting practices and children’s movement behaviors in LMIC communities. Therefore, the aims of this study were two-fold: (1) report the proportion of preschool children from low-income families in rural Brazil meeting the WHO 24-hour movement guidelines; and (2) determine associations with theory-based parenting practices related to physical activity, screen time, and sleep.

**Methods:**

A representative sample of 324 families from a rural district in north-eastern Brazil completed a validated, culturally adapted interviewer-administered survey assessing child physical activity, screen time and sleep, and associated parenting practices. The proportion of children meeting the physical activity, screen time, and sleep recommendations and all combinations of these recommendations was calculated. Forward selection logistic regression was used to determine which parenting practices were associated with meeting the individual recommendations and the 24-hour movement guidelines.

**Results:**

Less than half of the children (47.5%) met the physical activity recommendation, 22% met the screen time recommendation, 62% met the sleep recommendation, with just over 10% meeting all three recommendations in the 24-h movement guidelines. Having fewer rules and restrictions around indoor and outdoor play, limiting, or monitoring screen time, and maintaining a consistent bedtime routine were key parenting practices associated with children´s adherence to the 24-hour movement guidelines.

**Conclusion:**

Only 1 in 10 rural Brazilian preschool children meet the 24-hour movement guidelines. These findings underscore the need for family-based interventions targeting parenting practices to support healthful movement behaviors in young children from LMICs.

**Supplementary Information:**

The online version contains supplementary material available at 10.1186/s12966-022-01369-y.

## Background

The optimal combination of physical activity, sedentary time, and sleep is essential for health and development in early life [1]. Regular physical activity, reduced sedentary screen time, and sufficient sleep are associated with improved motor and cognitive development, psychosocial and cardiometabolic health, skeletal health, in addition to reducing the risk of overweight or obesity [1–4]. On the basis of this evidence, the World Health Organization (WHO)[5], along with public health organizations from Australia, Canada, South Africa, and the United Kingdom have issued 24-hour movement guidelines [6–9]. These guidelines recommend that children spend a minimum of 3 h a day in active play, with 60 min of moderate to vigorous physical activity; accumulate less than 60 min per day of sedentary screen time; and obtain between 10 and 13 h of good quality sleep every day [5–7]. In Brazil, physical activity guidelines for children under 5 years of age have been released and the recommendations for physical activity and sedentary screen time are the same as the 24-hour movement guidelines [10] .

Currently, little is known about the proportion of children meeting these recommendations in South American low-and middle-income countries (LMICs), such as Brazil. To date, only one study has evaluated adherence to the 24-hour movement guidelines in a sample of Brazilian preschoolers. de Lucena Martins et al. [11] measured the physical activity, screen time, and sleep behaviors of 270 Brazilian preschool children from the coastal city of João Pessoa, Brazil. The results indicated that 43% met the physical activity guideline, 15% met the screen time guideline, and 35% met the sleep guideline. Only 3% of children met all three of the recommendations included in the WHO 24-hour movement guidelines. While the results provided important information about adherence to the 24-hour movement guidelines among urban Brazilian preschool children, the extent to which preschool children residing in rural communities meet the 24-hour movement guidelines has not been evaluated. Importantly, young children residing in rural areas of Brazil are disproportionately affected by overweight and obesity [12] and are at increased risk of developing preventable long-term health conditions including coronary heart disease, diabetes, hypertension, depression, and certain cancers [13, 14]. Therefore, from a life course health perspective, it is essential to monitor the establishment of healthful movement behaviors during the first 2000 days of life [15].

Parents and caregivers play a central role in the development of children’s movement behaviors [16, 17]. Parenting practices are context-specific strategies and behaviors that parents use to assist or support children to achieve their socialization goals, including the establishment of healthy physical activity, screen time, and sleep behaviors [16, 18]. To date, a range of parenting practices have been investigated with logistic support, co-participation, modelling, and setting rules and limits emerging as significant correlates of children’s movement behaviors [19–22]. However, the bulk of this research has been conducted in families from high income countries (HICs) and little is known about the relationship between parenting practices and children’s movement behaviors in LMIC communities. Moreover, no previous study has examined associations between theory-based parenting practices and adherence to the 24-hour movement guidelines in children residing in a LMIC like Brazil. Importantly, a better understanding of how parenting practices influence child health behaviors is vital to inform family-based interventions targeting all three movement behaviors. Therefore, the aims of this study were two-fold: (1) determine the proportion of preschool-aged children from low-income families in rural Brazil meeting the WHO 24-hour movement guidelines, individually, and in combination; and (2) examine associations with theory-based parenting practices related to physical activity, screen time, and sleep and adherence to the 24-hour movement guidelines, individually and/or in combination.

## Methods

### Participants and setting

Parent-child dyads were recruited from 15 Early Childhood Education and Care Centers (ECECs) in Caruaru, Brazil. Caruaru encompasses a territorial area of 921 km^2^, representing 0.94% of the area of Pernambuco State. In 2021, it had a resident population of 369,343 inhabitants and a population density 342 people per square kilometer. The main economic activities in Caruaru are agriculture, tourism, and textiles and the region is responsible for about 30% of all garment production in Pernambuco. The average monthly salary of workers in Caruaru is 1.6 times the minimum wage in Brazil or approximately USD 320 per month, with approximately one-third of workers in the region earning less than half the minimum wage [23].

ECEC’s were randomly selected from three rural school districts using the Department of Education database as a sampling frame. Prior to selection, the sample was stratified by school district, and ECECs within each stratum were randomly sampled with a probability proportional to the total number of ECECs operating in each district. In Brazil, over 90% of children aged 5 years and under attend child care services.[24].

Participants were recruited in two ways. Prior to the COVID-19 pandemic, the Director from each ECEC was contacted by the principal investigator to explain the research and obtain consent for the center to participate in the study. If consent was given, a flyer was distributed to all parents of children aged 3- to 6-years inviting them to attend a meeting to explain the research project in detail. At the conclusion of this meeting, participant information sheets and consent forms were distributed. Parents who agreed to participate returned a signed informed consent form to the principal investigator. For parents with low literacy levels, verbal consent was obtained. During the COVID-19 Pandemic, when ECECs were closed, the Caruaru Department of Education sent an email to Directors inviting them to be contacted by the principal investigator and receive information about the study. ECEC Directors consenting to participate in the study then distributed information about the study to all families enrolled in the center via the Center’s Facebook page or WhatsApp. Parents interested in participating in the study gave permission to the Directors to share their contact information with the principal investigator. The principal investigator subsequently contacted the parent by phone to obtain informed consent and arrange a date and time for a phone interview. All recruitment and data collection activities were completed between October 2019 to July 2021. Importantly, over 90% of households in the northeast of Brazil have a mobile phone and over 98% use their device to access messaging and social media apps [25]. The research was approved by the Human Research Ethics Committee of the Queensland University of Technology, Brisbane, Australia (Approval No. 1,800,001,141), and the Department of Education of Caruaru, Brazil (Approval Letter March 1, 2019).

### Protocol

Parents completed an interview-administered survey measuring sociodemographic information, child physical activity, screen time, and sleep behaviors; and theory-based parenting practices related to physical activity, screen time, and sleep. Parents with multiple children enrolled in the ECEC completed the survey in relation to their first-born child. Prior to the COVID 19 pandemic, parents completed the survey as a face-to-face interview. However, during the COVID 19 pandemic, when ECECs were closed, parents completed the survey as a telephone interview. All interviews were conducted by the principal investigator. The survey took approximately 45 to 60 min to complete. Participating families received an inexpensive gift (e.g., soccer ball, skipping rope or Peteca; ~ USD $3.70 in value) in return for their time and effort.

### Measures

The following socio-demographic information was collected: child’s sex, date of birth, ethnicity, attendance at ECEC (part-time vs. full-time), caregivers’ age and gender, level of education, marital status, current employment status, household income, financial support from the government ‘Bolsa Família Programme’ and number of residents at home.

### Physical activity

Children’s physical activity was measured using the Burdette outdoor playtime recall [26], translated and culturally adapted for use in Brazilian families [27]. Parents reported the amount of time their child spent playing outdoors on a typical weekday and a typical weekend day in the last month. Scores were calculated using a weighted average of weekday and weekend reports. In a Brazilian sample of pre-school children, this measure exhibited strong evidence of test-retest reliability (ICC = 0.96) and concurrent validity with device-measured total movement (rho = 0.44, p < .05) and energetic play (rho = 0.39, p < .05) [28].

### Screen time

Child screen time was assessed using items from the Australian InFANT study [29], translated and culturally adapted for use in Brazilian families [27]. Parents reported their child’s screen time on a normal weekday and weekend day based on several digital media devices, including watching television programs, DVDs, using a computer, playing with an electronic game system (e.g., Nintendo DS, PlayStation, Xbox), and using smartphones, iPads, or Tablets. Scores were calculated using a weighted average of weekday and weekend reports. In a Brazilian sample of pre-school children, this measure exhibited strong evidence of test-retest reliability (ICC = 0.94), and construct validity based on device-measured sedentary time; (rho = 0.26, p < .05), total movement (rho = − 0.41, p < .05) and energetic play (rho = − 0.37, p < .05) [28].

### Sleep

Night-time sleep duration was measured using items adapted from the Prevention of Overweight in Infancy (POI) randomized control trial [30]. Parents reported the time their child usually went to bed at night and the time the child woke up in the morning to start the day on weekdays and weekend days, respectively. Scores were calculated using a weighted average of weekday and weekend reports. In a Brazilian sample of pre-school children, this measure exhibited strong evidence of test-retest reliability (ICC = 0.93), and concurrent validity with device-measured sleep duration (rho = 0.29, p < .05) [28].

### Parenting practices

Parenting practices related to physical activity and screen time were measured using scales developed by Vaughn and colleagues [31] - culturally adapted and validated for use among Brazilian families [27, 28]. These scales measure theory-based parenting practices used to control or support children’s physical activity and screen time behaviors. Controlling parenting practices included rules around active play indoors, rules around active play outdoors, use of physical activity to reward/control behavior, limiting outdoor play due to weather, limiting or monitoring of screen time, and use of screen time to reward/control child behavior. Supportive parenting practices included explicit modelling and enjoyment of physical activity, verbal encouragement for physical activity, importance and value of physical activity, exposure to screens, and explicit modelling and enjoyment of screen time. In a Brazilian sample of pre-school children, these scales presented evidence of acceptable internal consistency (McDonald’s Omega = 0.71–0.89) and test-retest reliability (ICC = 0.82–0.99) [28].

Parenting practices related to sleep were measured using four scales adapted from the Bedtime Routines Questionnaire [32]. The scales measured consistency of bedtime routine, sleep environment, reactivity to changes in sleep routine, and adaptive activities prior to bedtime. In a Brazilian sample of pre-school children, these scales exhibited evidence of acceptable internal consistency (McDonald’s Omega = 0.68–0.82) and test-retest reliability (ICC = 0.86–0.91) [28].

### Data reduction and analysis

Adjusted means and 95% confidence intervals for the child behaviors and parenting practices were estimated using general linear models. Each model included child sex, child age, and interview mode (face-to-face vs. phone) as fixed effects. Differences by child sex and interview mode were tested for statistical significance using single degree freedom contrasts with Bonferroni correction for multiple comparisons. The proportion of children meeting the physical activity (≥ 180 min/day), screen time (≤ 1 h/day), and sleep duration (< 3 years: 11 to 14 h/night; ≥ 3 years 10 to 13 h/night) guidelines and all combinations of these guidelines was calculated [5]. Forward selection logistic regression was used to determine which parenting practices were associated with meeting the individual guidelines and the 24-hour movement guidelines. Step 1 involved the inclusion of parental age, parental education, parental income, child age, and child gender as covariates, with parenting practices meeting the specified significance level entering the model in subsequent steps. All statistical procedures were performed using SAS statistical software version 9.4.

## Results

Socio-demographic data for participating families are presented in Table 1. Of the 625 families attending the 15 ECECs, 324 (52%) parent child-dyads consented to participate in the study and completed an interview. Participation rates across the 15 ECECs ranged from 33 to 92%. Of the 324, 100 (31%) parents completed the survey as a face-to-face interview, with the remaining 224 parents (69%) completing the survey as a telephone interview. As shown in Table 1, the socio demographic characteristics for the face-to-face interviews and the telephone interviews were similar, with no meaningful differences between the two groups.

**Table 1:** Socio-demographic characteristics of the whole sample and families completing the interview face-to-face or via telephone

Variables	Whole Sample N (%)	Face-to Face Interview N (%)	Telephone Interview N (%)
***Caregiver***			
Female	306 (95)	95 (97)	211 (93)
**Age** (years)			
≤ 24	67 (21)	16 (16)	51 (23)
25–35	165 (51)	52 (53)	113 (50)
> 36	92 (28)	30 (31)	62 (27)
**Marital status**			
Single parent family	139 (43)	44 (50)	95 (42)
Two adult caregiver	185 (57)	54 (50)	131 (58)
**Employment status**			
Employed full-time	63 (19)	20 (21)	43 (19)
Employed part-time	84 (26)	19 (19)	65 (29)
No paid work	177 (55)	59 (60)	118 (52)
Household income*			
<= 1 wage	281 (87)	88 (90)	193 (85)
> 1 wage	43 (13)	10 (10)	33 (15)
**Level of education**			
No school/ some elementary school	185 (57)	53 (54)	132 (58)
Completed elementary school/ some high school	71 (22)	25 (26)	46 (20)
Completed high school	60 (19)	16 (16)	44 (20)
Completed/incomplete tertiary education	8 (2)	4 (4)	4 (2)
**Number of residents in home**			
≤ 3	118 (36)	35 (36)	83 (37)
> 4	206 (64)	63 (64)	143 (63)
**‘Bolsa Família’ programme** ^**#**^	247 (76)	76 (78)	171 (76)
***Children***			
Female	170 (53)	54 (55)	116 (51)
Age in months (Mean ± SD)	62 ± 11	65 ± 8	61 ± 12
**Ethnicity**			
Caucasian or East Asian	123 (38)	41 (42)	82 (36)
Mixed-race, Afro Brazilian or Indigenous	201 (62)	57 (58)	144 (64)
**Attending child care**			
Half-time (~ 4 h/day)	272 (84)	100 (100)	174 (77)

* 1 wage was equivalent to R$997 monthly in Brazilian Real in 2019 (equivalent 190 USD); ^#^ Government assistance program for low-income families

Means and 95% confidence intervals for child physical activity, screen time, sleep, and associated parenting practice scales are reported in Table 2. On average, children were engaged in physical activity for 177 min per day, screen time for 181 min per day, and slept for 609 min (10.2 h) per night. When examined by child sex and interview mode, very few significant differences were identified. Scores on the limiting outdoor play due to weather and reactivity to changes in sleep routine scales were significantly lower in boys than in girls (p < .05). Scores on the rules around active play indoors, limiting outdoor play due to weather, use of screen time to reward/control child behavior, and reactivity to changes in bedtime routine scales were significantly lower for parents completing the telephone interview than those complete the face-to-face interview.

**Table 2:** Means and 95% confidence intervals for parent reported child health behaviors and parenting practices scales

	*Total Sample* *(N = 324)*	*Girls* *(N = 170)*	*Boys* *(N = 154)*	*Face to Face Interview* *(N = 100)*	*Phone Interview* *(N = 224)*
***Children’s movement behaviors***					
Physical Activity (mins/day)	177 (166–188)	168 (152–184)	184 (167–201)	173 (153–193)	179 166–193
Screen time (mins/day)	181 (166–196)	177 (156–199)	185 (162–208)	179 (152–207)	182 (164–201)
Night Sleep (mins/day)	613 (603–622)	613 (601–628)	615 (602–628)	619 (602–634)	610 (600–620)
***Controlling parenting practices***					
Rules around active play indoors	1.78 (1.72–1.84)	1.86 (1.77–1.94)	1.74 (1.65–1.83)	1.86 (1.76–1.97)	1.73 * (1.66–1.81)
Rules around active play outdoors	4.04 (3.90–4.18)	4.13 (3.93–4.33)	3.93 (3.72–4.15)	4.03 (3.77–4.29)	4.04 (3.87–4.21)
Use of physical activity to reward/control child behavior	3.32 (3.17–3.47)	3.28 (3.06–3.49)	3.45 (3.22–3.68)	3.47 (3.20–3.73)	3.26 (3.08–3.44)
Limiting outdoor play due to weather	2.87 (2.74–3.00)	3.09 (2.91–3.28)	2.78 # (2.58–2.98)	3.13 (2.89–3.36)	2.75 * 2.59–2.90)
Limiting or monitoring of screen time	3.29 (3.14–3.44)	3.21 (3.00–3.43)	3.41 (3.18–3.63)	3.34 (3.07–3.60)	3.28 (3.10–3.46)
Use of screen time to reward/control child behavior	4.41 (4.25–4.57)	4.48 (4.25–4.70)	4.57 (4.32–4.81)	4.81 (4.52–5.10)	4.23 * (4.04–4.43)
**Supportive parenting practices**					
Explicit modeling and enjoyment of physical activity	2.70 (2.61–2.80)	2.67 (2.54–2.80)	2.77 (2.63–2.90)	2.76 (2.60–2.93)	2.68 (2.57–2.79)
Verbal encouragement for physical activity	4.07 (3.95–4.19)	4.06 (3.89–4.23)	4.13 (3.95–4.31)	4.16 (3.95–4.37)	4.04 (3.89–4.18)
Logistic support for active play	2.81 (2.66–2.96)	2.71 (2.50–2.92)	2.94 (2.71–3.16)	2.84 (2.57–3.11)	2.80 (2.63–2.98)
Importance and value of physical activity	4.59 (4.53–4.64)	4.54 (4.46–4.63)	4.64 (4.56–4.73)	4.60 (4.50–4.71)	4.58 (4.52–4.65)
Exposure to screens	4.30 (4.07–4.53)	4.43 (4.11–4.76)	4.13 (3.78–4.47)	4.26 (3.85–4.67)	4.30 (4.03–4.58)
Explicit modeling and enjoyment of screen time	3.82 (3.73–3.92)	3.92 (3.78–4.05)	3.73 (3.59–3.88)	3.84 (3.66–4.00)	3.81 (3.69–3.92)
**Sleep parenting practices**					
Bedtime routine	4.10 (3.99–4.20)	4.07 (3.92–4.22)	4.05 (3.89–4.21)	3.97 (3.78–4.16)	4.15 (4.03–4.28)
Sleep environment	4.15 (4.06–4.23)	4.15 (4.02–4.27)	4.14 (4.01–4.28)	4.14 (3.98–4.30)	4.15 (4.04–4.25)
Reactivity to changes in bedtime routine	1.96 (1.85–2.07)	2.22 (2.07–2.37)	1.95 # (1.79–2.11)	2.43 (2.24–2.61)	1.75 * (1.62–1.87)
Adaptive activities prior to bedtime	3.82 (3.74–3.90)	3.83 (3.72–3.95)	3.83 (3.71–3.96)	3.87 (3.73–4.02)	3.79 (3.70–3.89)

# = Significant gender difference p ≤ .05; * = Significant differences for mode of interview

**Fig. 1:**
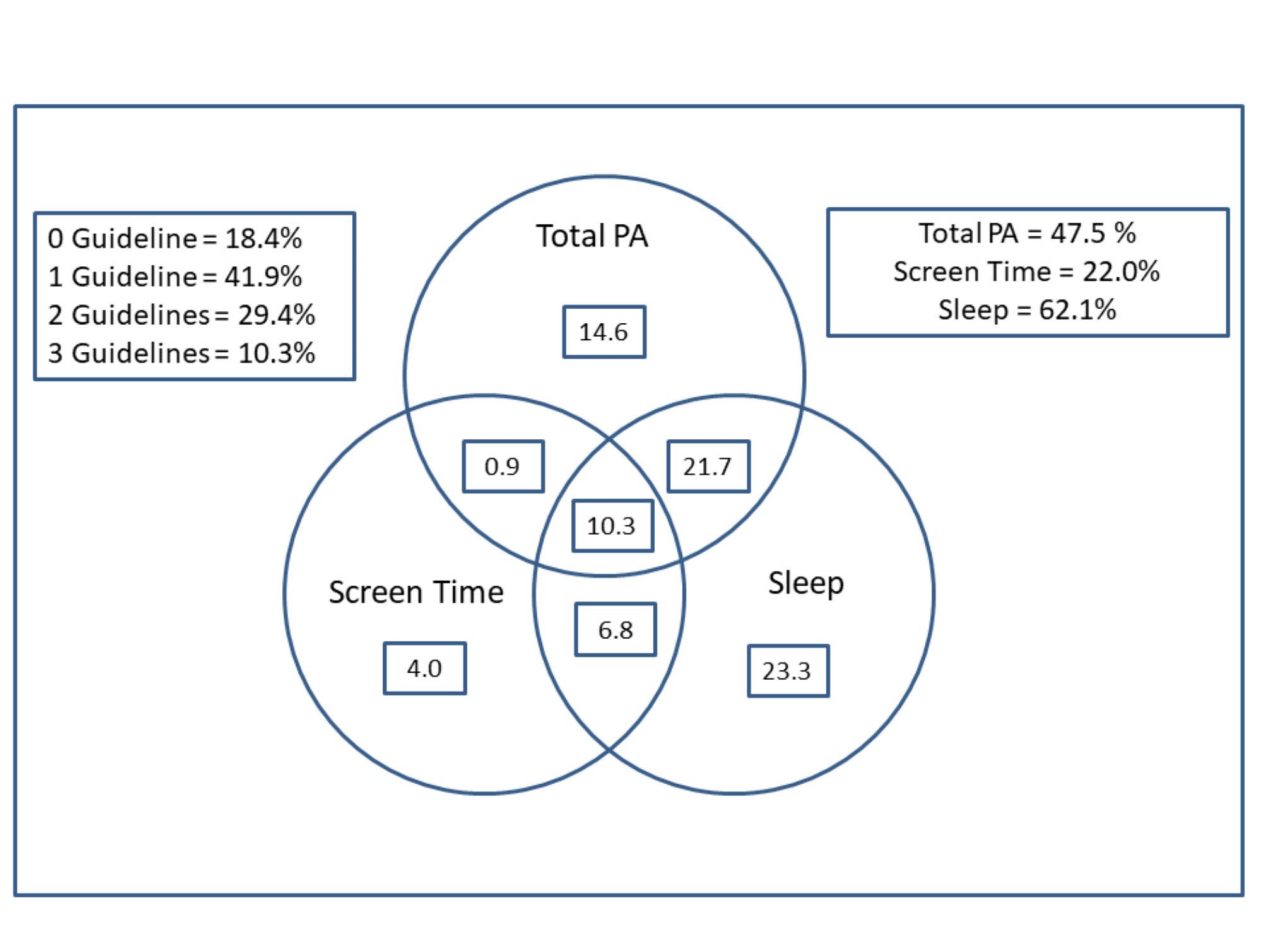
Venn diagram showing the proportion (%) of children meeting the physical activity, screen time, and sleep duration recommendations, and combinations of these recommendations

Figure 1 illustrates the proportion of children meeting the physical activity, screen time, and sleep duration recommendations, and combinations of these guidelines. Less than half of the children (47.5%) met the physical activity guideline, 22% met the screen time guideline, and 62% met the sleep guideline. Approximately 42% of children met just one guideline, 29% met two guidelines, with just over 10% meeting all three recommendations in the 24-h movement guidelines. Just over 18% of children failed to meet any of the guidelines.

The results of the stepwise logistic regression analyses for meeting the physical activity recommendation are summarised in Table 3. After controlling for parental age, parental education, parental income, child age, and child gender, rules around active play indoors, use of physical activity to reward/control child behavior, limiting outdoor play during poor weather, and bedtime reactivity to changes in sleep routine emerged as significant parenting practices. A one unit increase in rules around active play indoors, use of physical activity to reward/control child behavior and limiting outdoor play during poor weather decreased the odds of meeting the 180 min per day physical activity recommendation by 18–46%. Conversely, a one unit increase in the scale measuring child bedtime routine increased the odds meeting the physical activity recommendation by 50%.

**Table 3:** Odds ratios for parenting practices associated with meeting the physical activity recommendation, adjusted for socio-demographic variables

Physical Activity Recommendation	Odds Ratio	95% CI	Wald Chi Square	P-Value
Parent’s age	0.71	0.50–1.01	3.63	0.05
Parent’s education	0.83	0.63–1.10	1.65	0.20
Parental income	0.40	0.24–0.69	10.9	0.01
Child gender	1.17	0.72–1.89	0.40	0.52
Child age	1.67	1.30–2.14	15.8	< 0.01
Rules around active play indoors	0.54	0.34–0.86	6.67	0.01
Use of PA to reward/control behavior	0.80	0.66–0.96	5.58	0.01
Limiting outdoor play due to weather	0.82	0.70–0.97	5.30	0.02
Bedtime routine	1.50	1.07–2.09	5.71	0.01

The results of the logistic regression analysis for meeting the screen time recommendation are presented in Table 4. After controlling for parental age, parental education, parental income, child age, and child gender, limiting or monitoring of screen time, the use of explicit modelling and enjoyment of screen time, and reactivity to changes in sleep routine, emerged as influential parenting practices. A one unit increase in limiting or monitoring of screen time increased the odds of child meeting 60 min per day of screen time recommendation by 65%. Conversely, a one unit increase in explicit modeling and enjoyment of screen time, and reactivity to changes in sleep routine decreased the odds of child meeting screen time recommendation by 44–52%.

**Table 4:** Odds ratios for parenting practices associated with meeting the screen time recommendation, adjusted for socio-demographic variables

Screen Time Recommendation	Odds Ratio	95% CI	Wald Chi Square	P-Value
Parent’s age	0.97	0.64–1.46	0.02	0.87
Parent’s education	0.73	0.50–1.06	2.78	0.09
Parental income	0.50	0.26–0.97	4.19	0.04
Child gender	1.00	0.56–1.80	0.00	0.99
Child age	0.99	0.74–1.32	0.00	0.96
Limiting/monitoring of screen time	1.35	1.07–1.71	6.55	0.01
Modeling/enjoyment of screen time	0.48	0.35–0.67	18.4	< 0.01
Reactivity to changes in sleep routine	0.66	0.48–0.91	6.39	0.01

The results of the logistic regression analysis for meeting sleep recommendation are presented in Table 5. After controlling for parental age, parental education, parental income, child age, and child gender, none of the parenting practices were associated with meeting the recommendation. However, when the p-value for entry to the model was relaxed to P < .20, bedtime routine emerged as an influential, albeit non-significant parenting practice. A one unit increase in the bedtime routine scale was associated with 19% increase in the odds of meeting the sleep recommendation.

**Table 5:** Odds ratios for parenting practices associated with meeting the sleep recommendation, adjusted for socio-demographic variables

Sleep Recommendation	Odds Ratio	95% CI	Wald Chi Square	P-Value
Parent’s age	0.77	0.54–1.08	2.22	0.13
Parent’s education	0.78	0.59–1.03	3.05	0.08
Parental income	0.80	0.52–1.24	0.97	0.32
Child gender	0.91	0.57–1.48	0.13	0.71
Child age	1.79	1.40–2.30	21.3	< 0.01
Bedtime routine	1.19	0.93–1.51	1.87	0.17

The results of the logistic regression analysis for meeting all 3 recommendations in the 24-hour movement guidelines are presented in Table 6. After controlling for parental age, parental education, parental income, child age, and child gender, rules around active play outdoors, limiting outdoor play due to weather, and explicit modeling and enjoyment of screen time, emerged as influential parenting practices. A one unit increase in rules around active play indoors, limiting outdoor play during poor weather, and explicit modeling and enjoyment of screen time decreased the odds of meeting the 24-hour movement guidelines by 28–46%.

**Table 6:** Odds ratios for parenting practices associated with meeting all 3 recommendations of the 24-hour movement guidelines, adjusted for socio-demographic variables

Meeting All 3 Recommendations	Odds Ratio	95% CI	Wald Chi Square	P-Value
Parent’s age	0.80	0.45–1.41	0.59	0.43
Parent’s education	0.60	0.33–1.05	3.16	0.07
Parental income	0.35	0.09–1.25	2.60	0.10
Child gender	1.31	0.59–2.89	0.45	0.50
Child age	1.65	1.09–2.50	5.65	0.01
Rules around active play outdoors	0.70	0.51–0.96	4.73	0.02
Limiting outdoor play due to weather	0.72	0.55–0.91	6.96	< 0.01
Modeling/enjoyment of screen time	0.54	0.35–0.83	7.94	< 0.01

## Discussion

To our knowledge, the current study is the first to report the proportion of rural Brazilian preschool children meeting public health recommendations for physical activity, screen time, and sleep. A major finding was that only 10% of rural Brazilian preschool children met the WHO 24-hour movement guidelines. Less than half of the children met the physical activity recommendation, one-fifth met the screen time recommendation, while approximately 60% met the recommendation for sleep duration. Notably, just over 18% of children failed to meet any of the recommendations in the 24-hour movement guidelines. These findings underscore the need for policy initiatives and health promotion programs to promote healthful movement behaviors in young children from rural Brazil.

The proportion of children meeting the physical activity recommendation was comparable to that reported for urban Brazilian preschool children, while adherence to the screen time and sleep recommendations was substantially higher [11]. These findings are consistent with the results of a recent study comparing the physical activity and screen time behaviors of rural and urban preschool children in Brazil [33]. In that study, rural and urban preschoolers exhibited similar levels of device-measured total physical activity and energetic play, but rural preschool children had almost 1-hour less parent reported daily screen time than their urban counterparts [33]. The higher adherence to the sleep recommendation observed among rural preschool children is consistent with the results of Rae et al. [34] who reported 24-hour sleep duration to be significantly longer among low-income South African rural preschool children than urban preschool children. The authors hypothesized that children living in rural areas may have fewer environmental constraints on their sleep, e.g., lower housing density, more room and/or less bed sharing, less noise and less light pollution. Alternatively, the lower adherence rates reported for urban preschool children may be attributable to subtle methodological differences in the assessment of nighttime sleep duration. In the de Lucena Martins study [11], parents reported the total number of hours their child slept on weekdays and weekends during the night. In the current study, parents reported the time their child usually went to bed at night and the time the child woke up in the morning to start the day on weekdays and weekend days, respectively.

Recognized parenting practices such as rules related to indoor and outdoor play, use of physical activity to reward/control child behavior, limiting or monitoring screen time, parental modelling, and enjoyment of screen time, and maintaining a consistent bedtime routine were significantly associated with adherence to the 24-hour movement guidelines, individually and/or in combination. These findings are consistent with the results of studies conducted in HICs [19–22] and have important implications for the design of family-based interventions. Parents and caregivers enforcing restrictive rules around indoor and outdoor play could be educated about the consequences of authoritarian parenting style and receive instruction on the use of autonomy supportive parenting practices [16–18]. Parents and caregivers could be encouraged to monitor their own screen time and become positive role models for healthy screen time behaviors. Where appropriate, parents and caregivers could receive guidance on how to make a family media plan delineating mutually agreed upon goals and rules about screen time. Finally, parents could be asked to monitor their child’s nighttime routine and keep a record of situations or events that disrupt their child’s bedtime routine. Childcare settings are well-situated to support initiatives to assist parents from socioeconomically disadvantaged rural communities adopt such parenting practices. In Brazil, over 90% of children aged 5 years and under attend child care services [24] and early childhood educators are viewed as trusted sources of information about parenting [35]. To advance knowledge in this area and improve adherence to the 24-hour movement guidelines, future studies could conduct workshops with parents, educators, health professionals, and other relevant stakeholders and co-design prototype parenting interventions to increase physical activity, limit sedentary screen time, and improve sleep patterns in children aged 5 years and under.

Prior to the COVID 19 pandemic, parents completed the survey as a face-to-face interview. However, during the COVID 19 pandemic, when communities were in lockdown, parents completed the survey as a telephone interview. This provided an opportunity to explore the potential impacts of the COVID-19 lockdown on children’s movement behaviors. When the survey responses to the face-to-face and telephone interview were compared, there were no significant differences for child physical activity, screen time, and sleep. This suggests that, in this sample of rural Brazilian families, the COVID 19 lockdown had minimal impact on children’s movement behaviors. This result is consistent with the study conducted by Okely et al. [36] in which the physical activity and screen time behaviors of young children from LMICs were significantly less impacted by COVID-19 lockdowns than young children from HIC communities. There are a several possible explanations for this. First, preschool children engage in mostly unstructured free play activities which are not directly impacted by lockdowns, ECEC closures, and social-distancing restrictions. Second, the children in our study were not required to participate in distance education during lockdown, so there were no increases in screen time associated with mandatory online learning. Third, because parents can only report on the time they are with their child, proxy reports of children’s movement behaviors may not have captured changes in active play or screen time (positive or negative) resulting from ECEC closures during lockdown.

Within our sample, the odds of meeting guidelines for physical activity, screen time, and sleep decreased as the level of parental education and household income increased. Similar results were reported for Brazilian 5th grade students in relation to meeting public health guidelines for physical activity [37]. Our findings are also consistent with studies conducted South Africa in which preschool children from low-income families were found to have significantly lower levels of sedentary behavior, significantly higher levels of total physical activity, significantly less weekly screen time, and significantly longer 24-hour sleep duration than children from high-income families [34, 38]. Such findings support the concept that the relationship between socioeconomic status (SES) and movement behaviors is different in HICs and LMICs. In HICs, SES tends to be positively associated with physical activity while inversely related to sedentary behaviors such as screen time. But in LMICs, higher SES is associated with lower levels of physical activity and higher levels of sedentary behavior [39, 40]. The reverse SES gradient observed in LMICs could be explained, at least in part, by more affluent families having the means to purchase screen-based digital devices and services such as subscription-based television and streaming platforms, smartphones, tablets, and game consoles. It is also possible that low SES families from LMICs may be more reliant on active transport such as walking to get from place to place [40].

The current study has several strengths. It is the first study investigating adherence to the 24-hour movement guidelines and associated parenting practices in a representative sample of socioeconomically disadvantaged families from rural Brazil. Second, parenting practices and children’s physical activity, screen time, and sleep behaviors were assessed using validated measurement tools, culturally adapted for use among Brazilian families [27, 28]. Offsetting these strengths were several limitations. First, the study was cross-sectional in design, and therefore, causal relationships between parenting practices and children’s physical activity, screen time and sleep behaviors cannot be inferred. Future studies should employ prospective study designs to establish causal relationships between parenting and children´s movement behaviors. Second, participants were recruited from a single rural region in north-eastern Brazil, and the results may not be generalizable to other rural Brazilian communities. Finally, only a small number of father’s completed the survey; however, this is common in observational studies of parenting practices [41].

## Conclusion

In conclusion, only 1 in 10 rural Brazilian preschool children meet the WHO 24-hour movement guidelines, highlighting the need for policy initiatives and health promotion programs to establish healthy movement behaviors in young children from LMICs. Having fewer rules and restrictions around indoor and outdoor play, limiting or monitoring screen time, and maintaining a consistent bedtime routine were key parenting practices associated with meeting recommendations for physical activity, screen time and sleep. Family-based interventions to establish healthy lifestyle behaviors in preschool-aged children should target these parenting practices.

## Electronic supplementary material

Below is the link to the electronic supplementary material.

Supplementary Material 1 STROBE Statement–checklist of items that should be included in reports of observational studies

## Data Availability

The dataset supporting the conclusions of this article can be made available upon request after approval by the authors. Please direct inquiries to s.trost@uq.edu.au.

## Abbreviations

ECECs: Early Childhood Education and Care Centers.
HICs: High-income country.
LMICs: Low-middle income countries.
SES: Socioeconomic status.
Total PA: Total physical activity.
WHO: World Health Organization.
